# Evolution of *MUC1* During Retrotransposon Expansion as a Potential Adaptation Exploited in Human Cancer

**DOI:** 10.3390/ijms27146135

**Published:** 2026-07-09

**Authors:** Naoki Haratake, Shinkichi Takamori, Keisuke Shigeta, Donald Kufe

**Affiliations:** Department of Medical Oncology, Dana-Farber Cancer Institute, Harvard Medical School, 450 Brookline Avenue, D830, Boston, MA 02215, USAshinkichi_takamori@dfci.harvard.edu (S.T.);

**Keywords:** *MUC1*, M1C, IFN-I, HERV, LINE-1, *APOBEC3*, viral mimicry

## Abstract

The *MUCIN 1* (*MUC1*) gene evolved in eutherian mammals in association with the marked expansion of endogenous retroviruses (ERVs). *MUC1* encodes the MUC1-C/M1C protein that protects barrier epithelia from exogenous viruses. Activation of M1C in response to loss of homeostasis induces STAT1 and the type I interferon (IFN-I) pathway. Studies in cancer cells have found that M1C regulates human ERV (HERV) expression by a STAT1-mediated mechanism. These discoveries have uncovered new insights into M1C-induced regulation of HERVs and other retrotransposons, such as LINE-1 (L1) and Alu. M1C signaling integrates retrotransposon transcription with induction of the counteracting *apolipoprotein B mRNA-editing catalytic 3* (*APOBEC3*) genes that, like *MUC1*, first appeared in placental mammals. Activation of retrotransposons induces viral mimicry characterized as an IFN-I response that promotes innate anti-tumor immunity. Conversely, M1C protects cancer cells by sustained induction of the IFN-I pathway and immune evasion. This review posits that M1C-dependent regulation of retrotransposon and *APOBEC3* expression represents an adaptive response exploited by cancer cells that promotes malignant progression.

## 1. Introduction

### 1.1. Evolution of ERVs in Vertebrates

Vertebrates have served as hosts for retroviruses dating back hundreds of millions of years [1]. Retroviruses have been propagated throughout vertebrate evolution by integration of proviruses into host germline cells. The human genome contains about 700,000 loci of sequences that are potentially derived from retroviruses [1].

Most human endogenous retroviruses (HERVs) are defective as a result of having accumulated deletions and other mutations during vertebrate evolution [1,2]. Other evolutionarily younger HERVs, such as HERV-K(HML-2), have intact viral genomes with 5′-LTR and 3′-LTR regulatory sequences and *gag*, *pro*, *pol* and *env* genes [1]. Together, the ancient and more recently acquired HERVs account for ~8% of the human genome [3].

### 1.2. HERVs in Evolution and Cancer

Retroviruses and their hosts have exchanged genes throughout evolution [4]. A notable example is the Rous sarcoma virus in which *v-src* exapted from the host *src* gene conferred the capacity for inducing uncontrolled proliferation and the formation of sarcomas [5]. This discovery led to the identification of multiple other oncogenes that were initially uncovered in retroviruses and later found in host cells [6].

ERVs have contributed to the evolution of humans by functioning as agents of selection and genetic diversity [1,7,8,9]. HERVs confer a selective advantage by playing important roles in mammalian reproductive fitness [10]. For example, the HERV *env* gene encodes Env glycoproteins necessary for formation and function of the eutherian placenta [11,12,13,14].

On the other hand, HERVs promote oncogenesis by inducing DNA replication stress and genomic instability [9,10]. HERV Env proteins also contribute to cancer progression by driving lineage plasticity, proliferation and stemness across different tumor types [10]. HERVs thus represent an example of antagonistic pleiotropy in which genes that increase fitness for reproduction can contribute to cancer later in life [15,16].

The available evidence thus indicates that HERVs drive natural selection in evolution and cancer. However, a more precise understanding is needed of the putative pathways by which HERVs promote malignant progression.

## 2. MUC1 Evolved to Protect Against Exogenous Viruses

### 2.1. Appearance of the MUC1 Gene in Mammals

*MUC1* appeared in eutherian mammals during a period of marked ERV expansion approximately 30–40 million years ago [2,17]. *MUC1* is of importance in placental development and reproductive fitness [17]. *MUC1* also evolved to protect barrier epithelia from disruption of homeostasis by exogenous viruses and other biotic insults [17]. As reviewed recently in regard to a potential maladaptation of this protective function, *MUC1* promotes cancer progression and represents an example of antagonistic pleiotropy [17]. The present review focuses on another perspective that *MUC1* may contribute to cancer by playing a role in the regulation of HERVs and other retrotransposons.

*MUC1* encodes two subunits: (i) a MUC1-N extracellular mucin that provides a protective physical barrier and (ii) a MUC1-C/M1C transmembrane non-mucin protein that regulates the inflammatory response to viruses [17,18,19]. In this way and as selected examples, activation of *MUC1* limits the severity of Influenza A Virus (IAV) [20], Respiratory Syncytial Virus (RSV) [21], HIV-1 [22], and SARS-CoV-2 [23] infections.

M1C integrates the inflammatory response with activation of proliferative and remodeling pathways that are theoretically reversible with resolution of the viral insult [17]. However, in settings of chronic inflammation, persistent M1C activation imprints epigenetic changes that become heritable in promoting malignant transformation [17]. The available evidence thus indicates that M1C evolved to protect reproductive and barrier epithelia exposed to exogenous viruses [17]. We propose that this protective role may extend to HERVs with the potential capacity for driving chronic inflammation and cancer.

### 2.2. M1C Regulates the Innate Immune Response

The innate inflammatory response is induced by dsRNA-mediated activation of the MDA5 and RIG-I pattern recognition receptors (PRRs) [24,25]. Accumulation of DNA in the cytosol is recognized by activation of the cGAMP synthase (cGAS)-stimulator of the IFN (STING) pathway [26]. Stimulation of these sensors by cytosolic nucleotides induces production of type I IFNs [24,25,26].

Activation of HERVs results in transcription of dsRNAs that are detected by PRRs and induce the IFN-I pathway [27]. HERVs also contribute to DNA replication stress by inducing DNA double-stranded breaks, deletions and translocations [28,29,30]. Thus, in addition to forming dsRNA, HERVs induce genomic instability and thereby the cGAS/STING pathway [27].

**Figure 1:**
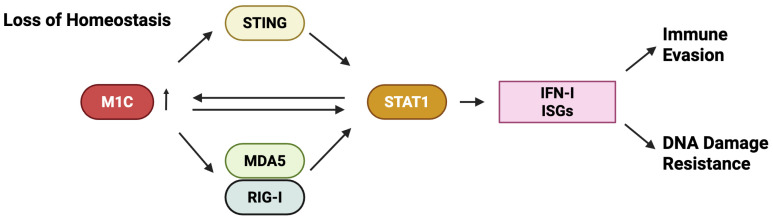
M1C-mediated activation of the IFN-I pathway in cancer cells. M1C is chronically activated by DNA replication stress in cancer cells. M1C activates STAT1 in an auto-inductive pathway that is amplified by treatment with targeted agents. In turn, M1C/STAT1 signaling drives expression of MDA5, RIG-I and STING, which are activated by genomic instability and induce ISGs that confer DNA damage resistance and immune evasion. [Created in BioRender. Haratake, N. (2026) https://BioRender.com/y6smkc1 (accessed on 1 July 2026)].

M1C is necessary for the induction of (i) MDA5, RIG-I, STING expression and (ii) IFN-I signaling in cancer cells (Figure 1) [17,31,32,33]. Chronic activation of M1C signaling induces STAT1 and other IFN-stimulated genes (ISGs) that confer DNA damage resistance and immune evasion (Figure 1) [17,31,32,33]. M1C sustains IFN-I signaling by binding directly to STAT1 and inducing STAT1 target ISGs (Figure 1) [17,34].

M1C and STAT1 also activate STING in an auto-inductive inflammatory pathway [17,19]. This “alarm” signaled by genomic instability is, in principle, reversible with resolution of the insult. However, in settings of prolonged DNA replication stress, chronic activation of the M1C-driven inflammatory response can become established by heritable epigenomic alterations [17,19].

## 3. M1C Regulates HERV Expression in Cancer Cells

### 3.1. Regulation of HERV Expression

HERV genomes are largely repressed in somatic cells by epigenetic mechanisms that often involve histone and DNA methylation [35]. HERV expression is thus increased in early embryonic stem cells (ESCs) by suppression of H3K9 trimethylation [35]. HERV transcription is also induced in cancer cells by treatment with DNA methyltransferase (DNMT) inhibitors [36,37].

Opposing mechanisms that suppress HERV expression reviewed below are of importance in controlling the inflammatory response to DNA replication stress. In this regard, dysregulation of HERV derepression is associated with chronic inflammation in cancer, autoimmune diseases and neurodegenerative disorders by pathways that largely remain unclear [38,39,40,41].

### 3.2. M1C Induces HERV-K Transcription in Cancer Cells

Genomic instability is a characteristic of malignant cells [42]. M1C is activated by DNA replication stress and induces the IFN-I pathway [17,19]. A previously unexplored area of investigation was whether the activation of M1C and the IFN-I pathway plays a role in the regulation of HERVs. This line of inquiry was therefore addressed in cancer cells with a focus on DNA replication stress induced by targeted agents [43,44].

In non-small cell lung cancer (NSCLC) EGFR mutant cells treated with the EGFR inhibitor osimertinib, M1C was found to be sufficient for the induction of HERV *gag*, *pol* and *env* expression [43]. This M1C-dependency was identified in activation of the evolutionarily younger HERV-K102(1q22) and HERV-K108(3q12.3) loci [43]. The HERV-K102 and HERV-K108 5′LTRs contain STAT1-binding motifs, which were found to be activated by M1C/STAT1 complexes [43]. The activation of these HERV-Ks was also dependent on STING, consistent with induction in response to DNA replication stress [43].

Treatment of castration-resistant prostate cancer (CRPC) cells with the PARP inhibitor olaparib confirmed that M1C is necessary for the induction of HERV-K102/108 *gag*, *pol* and *env* expression [44]. The demonstration that HERV-K102/108 activation is STAT1- and STING-dependent provided evidence that this M1C-induced pathway drives HERV-K expression in different types of cancer cells and in settings of treatment with distinct targeted agents.

### 3.3. M1C Regulates Env Translation in Cancer Cells

HERV-K Env is expressed on the surface of pluripotent stem cells and in the testes and ovaries [45,46]. The HERV env-encoded syncytins promote reproductive fitness of the placenta and, paradoxically, metastatic behavior of cancer cells [10,11,12,14]. Dysregulation of HERV-K Env expression is further associated with diverse cancers, as well as neurodegenerative and autoimmune diseases [47,48,49,50,51]. Of potential importance in this respect, M1C-driven induction of HERV-K *env* transcripts is associated with translation of the Env protein [44].

These findings uncovered a previously unrecognized M1C-dependent pathway responsible for the induction of HERV-K transcription and Env translation in human cancers.

## 4. M1C Inflammatory Signaling and Viral Mimicry

### 4.1. Viral Mimicry

**Figure 2:**
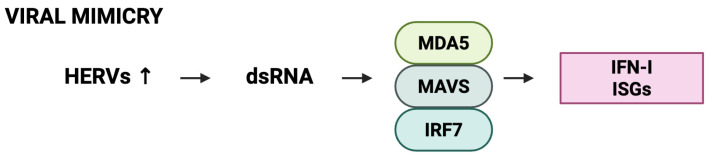
Activation of viral mimicry in cancer cells. Treatment of cancer cells with DNA methyltransferase (DNMT) inhibitors derepresses HERV transcription with the production of dsRNA. In turn, dsRNA stimulation of MDA5/MAVS/IRF7 signaling activates the expression of IFN-I ISGs. The “viral mimicry” response to HERV activation induces innate anti-tumor immunity. [Created in BioRender. Haratake, N. (2026) https://BioRender.com/y6smkc1 (accessed on 1 July 2026)].

Retroelements act as alarms that alert the cell to disruption of homeostasis by activating a viral mimicry response (Figure 2) [52,53,54]. Viral mimicry was initially identified in cancer cells as a response induced by DNA demethylating agents that derepress retroelements [36,37]. This response to HERV activation is driven by dsRNA stimulation of MDA5/MAVS/IRF7 signaling and induction of IFN-I ISGs (Figure 2) [36,37]. Subsequent work found that viral mimicry is induced by treatment of cancer cells with cytotoxic and targeted agents, indicating a potential link of this response to DNA replication stress (Figure 2) [52,53,54].

### 4.2. M1C Activation of the IFN-I Pathway Parallels Viral Mimiry

Viral mimicry activates the innate immune response and anti-tumor immunity (Figure 2) [52,53,54]. Paradoxically, activation of HERVs and viral mimicry is also associated with cancer progression [52,53,54]. The basis for this distinction has largely remained unclear despite numerous studies on the intersections of HERVs, innate immunity and cancer, which have provided the foundation for further discovery in this complex field.

**Figure 3:**
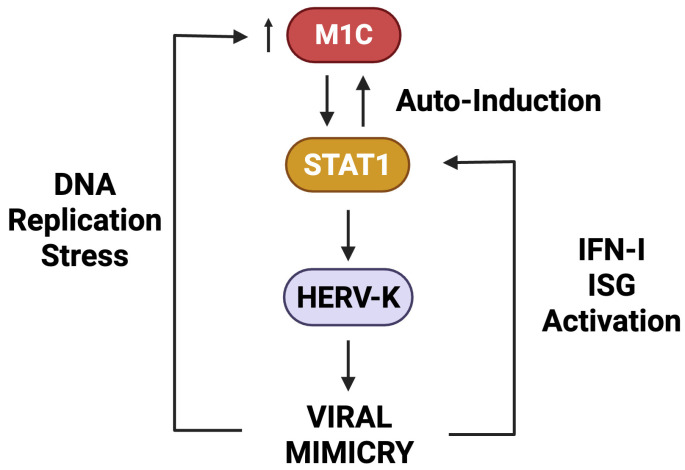
M1C/STAT1 signaling induces HERV-K transcription in potential chronic feedback pathways with viral mimicry. M1C is constitutively activated by DNA replication stress in cancer cells. M1C binds directly to STAT1 and forms an auto-inductive pathway that is amplified by targeted agents. M1C/STAT1 signaling activates HERV-K transcription, which induces viral mimicry and IFN-I ISGs with the potential for driving chronic feedback pathways and immune evasion. [Created in BioRender. Haratake, N. (2026) https://BioRender.com/y6smkc1 (accessed on 1 July 2026)].

One potential previously not entertained explanation is that sustained induction of viral mimicry could be associated with activation of the M1C/STAT1 auto-inductive pathway and chronic inflammation. In this regard, M1C induces HERV expression by activating STAT1/IFN ISG pathway that parallels the viral mimicry response (Figure 3). Induction of viral mimicry and the IFN-I pathway has been considered a way of converting tumors from “cold” to “hot” by recruiting immune effector cells into the microenvironment [36,37]. Conversely, the IFN-I pathway is potentially a “double-edged sword” in that chronic activation can confer immune evasion [55].

## 5. M1C Induces LINE-1 Expression in Cancer Cells

### 5.1. LINE-1 Activates the IFN-I Pathway

HERVs are grouped into the family of LTR retrotransposons. Non-LTR transposons include the (i) Long Interspersed Elements 1 (LINE-1s or L1s); (ii) Short Interspersed Elements (SINEs), such as Alus; and (iii) SINE-VNTR-Alus (SVAs). The evolutionary history of L1s in primates is far less well characterized than that for ERVs [2]. L1-derived sequences and the human-specific L1Hs (L1PA1) subfamily constitute ~17% of the human genome [56,57]. L1s play important roles in ESC self-renewal, preimplantation embryos and placental development [58,59].

L1s have open-reading frames 1 and 2 that encode the L1-ORF1p and L1-ORF2p proteins essential for L1 retrotransposition and the mobilization of non-autonomous retroelements [2]. L1-ORF1p is an RNA chaperone [60]. L1-ORF2p has reverse transcriptase and endonuclease activities that catalyze DNA integration during retrotransposition [61].

Activation of L1s results in the formation of dsRNA that triggers IFN-I signaling and viral mimicry [62]. L1 cDNA intermediates, RNA/DNA hybrids, as well as the L1-ORF1p protein [63], also function as viral mimics that activate the innate immune response. Additionally, L1s activate innate immunity through their mutagenic endonuclease activity, and thereby induction of STING and the IFN-I pathway [64].

### 5.2. M1C/STAT1 Signaling Induces LINE-1 Expression

**Figure 4:**
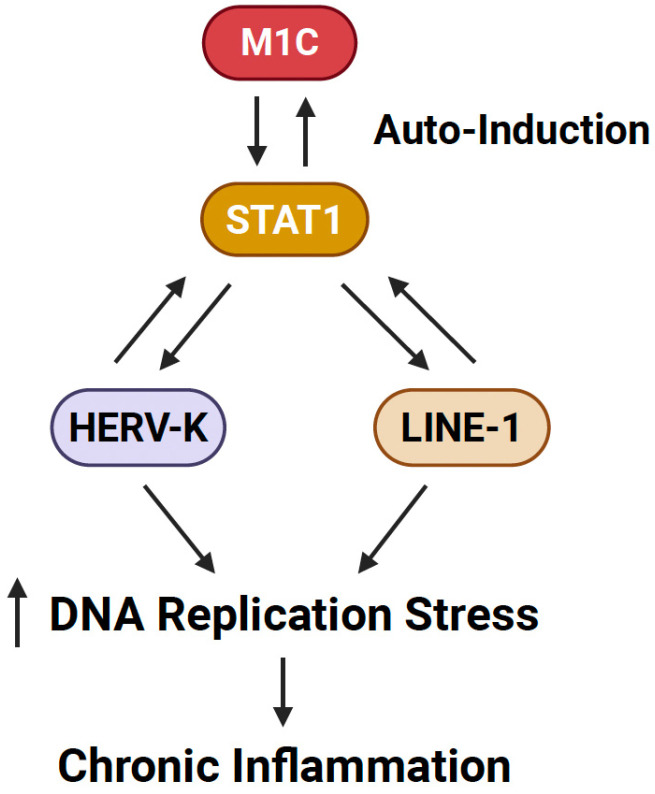
M1C activates HERV-K and LINE-1 retrotransposons in auto-stimulatory pathways that promote chronic inflammation. M1C activates HERV-K and L1 transcription by STAT1-dependent signaling that is proposed here to confer sustained DNA replication stress and chronic inflammation in auto-stimulatory pathways. [Created in BioRender. Haratake, N. (2026) https://BioRender.com/y6smkc1 (accessed on 1 July 2026)].

In cancer cells, M1C has been found to be necessary for constitutive expression of L1-5′UTR, L1-ORF1p and L1-ORF2p transcripts and for their induction by targeted agents (Figure 4) [44]. L1 mediates the transcription of SINEs, such as AluY, and along these lines M1C induces AluY expression [44]. In parallel with HERV-Ks, M1C drives L1-5′UTR, L1-ORF1p and L1-ORF2p expression by activation of the STAT1/STING pathway [44]. The L1-ORF1p protein is widely upregulated in human cancers by unclear mechanisms [65]. In this context, M1C/STAT1/STING signaling induces the L1-ORF1p protein in cancer cells [44].

Notably, L1 transcription, rather than retrotransposition, is sufficient for altering chromatin architecture and gene expression across cancer cells [66]. M1C is sufficient for L1 transcription, whereas studies will be needed to determine if M1C plays a role in L1 retrotransposition. These findings have collectively uncovered that M1C regulates HERV-K and L1 expression in cancer cells by activating STAT1 in association with the induction of IFN-I ISGs.

## 6. M1C Regulates Retrotransposon Expression by Inducing Counteracting Suppressive Pathways

Host genomes have evolved to suppress activation of retrotransposons as defense mechanisms to counteract potential detrimental effects of excessive mutagenic activity [67,68]. M1C activates HERVs and L1s; however, an unexplored question was whether M1C also drives other activating and counteracting pathways to control or “fine-tune” their expression?

Retrotransposons are repressed by mechanisms that include DNA and histone methylation. In cancer cells, M1C induces (i) DNMT1/3b expression and DNA methylation of L1 repeats [69,70], and (ii) the EZH2/PRC2 histone methyltransferase [71]. Given the findings reviewed here, additional studies are needed to determine if M1C regulates retrotransposon expression by DNA and/or histone methylation.

Opposing mechanisms that suppress retrotransposons include the (i) mammalian apolipoprotein B mRNA-editing catalytic 3 (*APOBEC3*) cytidine deaminases [72], (ii) Kruppel-associated box (KRAB) zinc-finger proteins [73] and (iii) human silencing hub (HUSH) epigenetic transcriptional repressor complex [74]. However, it was not known if M1C contributes to the regulation of these counteracting pathways.

### 6.1. APOBEC3 Cytosine Deaminases

The origin of *APOBEC3* genes in primates occurred concurrently with marked expansion of ERVs and L1s [2]. Intriguingly, like *MUC1*, the *APOBEC3* genes first appeared in placental mammals [75]. The encoded *APOBEC3* cytosine deaminases play roles in both protecting against exogenous viruses and counteracting the mobilization of ERVs and L1s [2].

The origin and expansion of APOBEC3s (A3s) in primates coincided with retrotransposon evolution in what has been described as an evolutionary arms race [68,72,76]. In addition to playing a protective role in counteracting retrotransposon activation, A3s have been linked to inducing (i) mutational signatures across cancer cell genomes, (ii) tumor heterogeneity and (iii) treatment resistance [77,78,79,80].

In humans, the *APOBEC3* gene family located on chromosome 22 consists of seven A3A/B/C/D/F/G/H members [2,72]. Of these, A3A and A3B are activated in cancer cells treated with targeted agents and thereby contribute to genomic instability and treatment resistance [81,82,83,84]. A3C and A3D also confer resistance to treatment-induced DNA replication stress [85].

Despite the co-evolution of *MUC1* and A3s in placental mammals and their corresponding roles in protecting against viral infections, there was no evidence that M1C functions in regulating A3 expression.

### 6.2. M1C Induces APOBEC3 Expression

Recent work has found that M1C is necessary for the induction of A3A expression and activity in cancer cells [43,44]. Silencing M1C and A3A enhanced the cytotoxic activity of targeted agents [43,44], consistent with the findings that A3A regulates the effects of treatment on cancer cell survival [83,86].

M1C was also identified as being essential for A3B, A3C, A3D and A3G expression [43,44]. A3B and A3D originated during L1 invasion [2,72]. The A3G locus appeared in humans around the time of peak HERV expansion [87]. A3G protects cancer cells from DNA damage and induces a unique mutational signature [88,89].

### 6.3. M1C Integrates Retrotransposon and APOBEC3 Expression

M1C-induced activation of HERV-K and LINE-1 transcription is conferred by unphosphorylated STAT1 that as homodimers form U-GAF complexes [43,44]. Unphosphorylated STAT1 also forms U-ISGF3 complexes with unphosphorylated STAT2 and IRF9 that drive expression of IFN-I ISGs [24,90].

**Figure 5:**
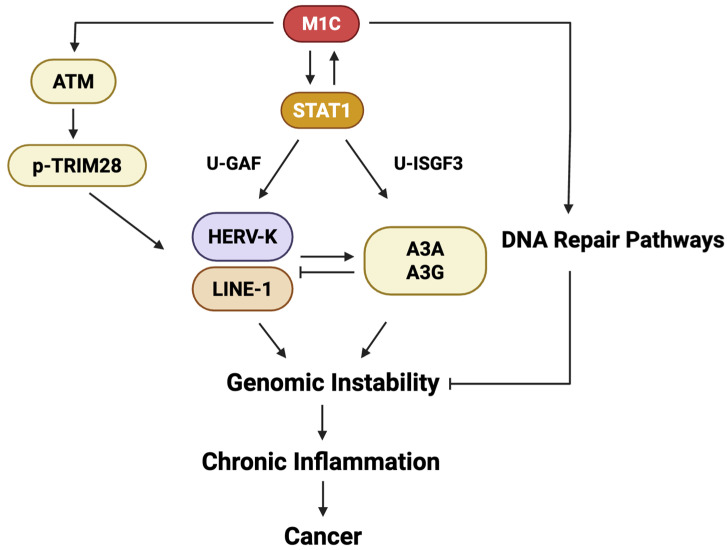
M1C integrates regulation of retrotransposons and A3s with DNA repair pathways with the potential for promoting survival of cancer cells. M1C induces A3A to counteract HERV-K and L1 expression. Conversely, M1C induces the expression of ATM, which phosphorylates KAP1/TRIM28 with the derepression of HERV-K and L1. The induction of retrotransposons and A3A, as well as other A3s, is mutagenic, necessitating activation of pathways to limit DNA replication stress and promote survival. M1C has the potential capacity for circumventing excessive DNA damage by inducing DNA repair pathways. [Created in BioRender. Haratake, N. (2026) https://BioRender.com/y6smkc1 (accessed on 1 July 2026)].

In parallel with M1C-driven induction of HERV-Ks and L1s by U-GAF complexes, M1C activates A3A expression by the STAT1/STAT2/IRF9 U-ISFG3 pathway [43,44]. M1C also induces other A3s, including A3G, by this pathway [43]. M1C signaling thereby has the capacity for integration of retrotransposon and A3 expression by STAT1 containing U-GAF and U-ISFG3 complexes (Figure 5) [43].

These findings provided support for the premise that coevolution of *MUC1* and A3s in placental mammals may have occurred in association with the invasion of HERVs and L1s. A3s counteract mobilization of these retrotransposons to protect against genomic instability, yet on the other hand, A3s are themselves mutagenic and oncogenic. Given these findings, it is conceivable that M1C integrates the extent of retrotransposon activation with their suppression by A3s in cancer cells to protect against excessive DNA replication stress and loss of survival (Figure 5).

### 6.4. M1C Activates DNA Repair Pathways as a Potential Way of Protecting Against Genomic Instability Induced by Retrotransposon and APOBEC3 Expression

ATM plays a pivotal role in orchestrating the DNA damage response [91]. M1C drives activation of the ATM gene by an E2F1-mediated mechanism and interacts directly with ATM in forming a complex with gH2AX [44,92]. M1C drives the (i) expression of other key DNA repair genes that include those encoding ATR, BRCA1/2, PALB2, CHEK2, FANCD2 and RAD51, and (ii) activation of DNA double-stranded break (DSB) repair and homologous recombination (HR) repair pathways (Figure 5) [44]. As a result, M1C has the capacity to function at the interface of regulating the DNA damage response and activation of the IFN-I pathway.

ATM phosphorylates the KRAB-associated protein 1 (KAP1/TRIM28) on S824 in promoting DNA repair [93,94]. Herein lies a provocative link between DNA damage and activation of retrotransposons in that (i) TRIM28 is a repressor of HERV and L1 transcription and (ii) ATM phosphorylation of TRIM28(S824) derepresses retrotransposon expression [44,95,96,97]. M1C is of potential importance at this interface by activating ATM-mediated TRIM28(S824) phosphorylation and derepressing retrotransposon transcription (Figure 5; Table 1) [44].

TRIM28 silences retrotransposon regulatory elements by binding to KRAB-zinc finger protein (KZNFs), recruitment of the SETDB1 histone methyltransferase and deposition of repressive H3K9me3-mediated heterochromatin [98]. TRIM28 cooperates with the HUSH complex that includes SETDB1 and functions in repressing ERVs and L1s [74]. HUSH is of particular interest as a potential M1C-regulated complex that also functions in activation of the IFN-I pathway [74].

In summary, M1C has the capacity to interface with several pathways that impact upon retrotransposon expression (Table 1). This role of M1C, which is emerging with other studies, lend credence to the notion that *MUC1* evolved during retrotransposon expansion as a way of contributing to their regulation.

## 7. Discussion

HERVs comprise ~8% of the human genome as ancient elements inactivated during vertebrate evolution and more recently integrated proviruses, such as HERV-Ks, with the capacity for transcription and translation [2]. L1-derived sequences constitute ~17% of the human genome and regulate the expression of SINEs/Alus [2]. The importance of retrotransposons in human evolution is a field of active investigation in which much remains to be known about the regulation of their expression and functions [99,100,101].

The *MUC1* gene appeared in eutherian mammals within the period of marked ERV and L1 expansion [2,17,19]. Work on *MUC1* since identification of the gene about 40 years ago by the Kufe laboratory focused initially on involvement of the encoded MUC1-N mucin and MUC1-C/M1C non-mucin subunits in pan-cancer progression [17,19]. The premise being that *MUC1* evolved to protect barrier tissues from loss of homeostasis and that this function is exploited by cancer cells for their survival [17,19].

Recent work reviewed here sheds new light on the natural selection of *MUC1* in mammals. HERVs play important roles in reproductive fitness and genetic diversity [1,7,10]. L1s also contribute genetic novelty to host genomes [56,102]. On the other hand, dysregulation of HERV and L1 expression, as for example in settings of chronic inflammation, may contribute to detrimental genomic instability, cancer and neurodegenerative disorders. We posit that (i) M1C evolved in mammals to regulate retrotransposons and (ii) this M1C function has been subverted by cancer cells to promote malignant progression.

## 8. Conclusions

Mammals have acquired the capacity for regulating the activation and suppression of retrotransposons. The evolution of *MUC1* in eutherians represents a potential adaptation that (i) regulates the induction of HERVs and L1s and (ii) counteracts their expression by activating A3s. This M1C-driven adaptation is integrated with activation of the IFN-I pathway, which we propose is exploited by cancer cells as a maladaptation of sustained viral mimicry and chronic inflammation that promotes DNA damage resistance and immune evasion.

The limitations of M1C involvement in regulating retrotransposons and APOBEC3s are that the work reviewed here was performed on cancer cells treated with targeted agents that induce DNA replication stress. We note that these findings may not be reflective of M1C and retrotransposon activation in reproductive and barrier tissues in the response to loss of homeostasis. These findings in cancer cells could also be associated with, but not contributory to, malignant progression, which will need to be addressed in subsequent studies.

## 9. Future Directions

Studies are needed to determine if M1C regulates other mechanisms that control retrotransposon expression, such as the (i) Piwi-interacting RNA (piRNA) pathway [103] and (ii) HUSH complex, which epigenetically silences retrotransposons and, interestingly, functions as a gatekeeper of the IFN-I pathway [74,104]. Future work will also be necessary to determine if, in addition to regulating retrotransposon transcription, M1C plays a role in retrotransposition. Work on *MUC1* has largely focused on involvement in driving pan-cancer progression, keeping in mind that this gene appeared in placental mammals and plays a role in reproductive fitness [17]. Further insight into the role of M1C in innate immunity will likely be uncovered by studies of the privileged maternal–fetal interface that has long been considered as a potential model for immune evasion in cancer.

## Figures and Tables

**Table 1:** Principal pathways regulating retrotransposons and APOBEC3s.

Pathway	Effect on Retrotransposons
DNA/histone methylation, KRAB-ZFP/KAP1/TRIM28/SETDB1 and HUSH	Epigenetic repression of ERVs/HERVs and LINE-1
DNMT inhibitor-mediated HERV derepression	HERV transcription, dsRNA formation, MDA5/MAVS/IRF7 signaling and IFN-I ISG induction
M1C/STAT1/U-GAF signaling	HERV-K and LINE-1 activation with potential viral mimicry, DNA replication stress and chronic inflammation
M1C/U-ISGF3-APOBEC3 signaling	APOBEC3A and APOBEC3G induction that counteracts HERV/LINE-1 mobilization, while contributing to mutagenesis
M1C/ATM-KAP1/TRIM28 signaling	KAP1/TRIM28 phosphorylation with potential derepression of HERV-K and LINE-1

## Data Availability

No new data were created or analyzed in this study. Data sharing is not applicable to this article.
